# Routine Neuroimaging in Patients with Stage IV Non-Small Cell Lung Cancer: A Single Center Experience

**DOI:** 10.3390/curroncol28020108

**Published:** 2021-03-02

**Authors:** Maude Dubé-Pelletier, Catherine Labbé

**Affiliations:** Institut Universitaire de Cardiologie et de Pneumologie de Québec, Université Laval, Laval, QC G1V 0A6, Canada; maude.dube-pelletier.1@ulaval.ca

**Keywords:** stage IV non-small cell lung cancer, central nervous system metastasis, neuroimaging

## Abstract

Background: There is a lack of consensus in current practice guidelines regarding routine neuroimaging in patients with stage IV non-small cell lung cancer (NSCLC) without neurologic symptoms, and there is a paucity of data on the impact of such imaging on overall survival (OS). Methods: This retrospective study included 257 patients with stage IV NSCLC without neurologic symptoms diagnosed between January 1, 2013 and December 31, 2016 at *Institut universitaire de cardiologie et de pneumologie de Québec* (IUCPQ). The primary objective of this study was to compare the evolution of patients with stage IV NSCLC who had baseline brain imaging versus with who did not. Secondary objectives were to determine the proportion of patients who underwent brain imaging in their initial investigation and the proportion of patients who developed metachronous central nervous system (CNS) metastasis. Results: CNS imaging, mainly with computed tomography (CT), was performed at diagnosis in 56% of patients, and the prevalence of synchronous CNS metastasis among these patients was 32%. There was no difference in median OS between patients who underwent initial CNS imaging and those who did not, but we did show a tendency for a higher cumulative incidence of metachronous CNS metastasis in patients without baseline imaging. These metachronous metastases were symptomatic and were more often not treated when compared to synchronous metastases. Conclusions: In this small, unicentric retrospective study, there was no benefit with routine neuroimaging in terms of median OS in stage IV NSCLC patients without neurologic symptoms.

## 1. Introduction

Lung cancer, specifically non-small cell lung cancer (NSCLC), is the most common primary malignancy that metastasizes to the brain [1]. In several prospective and retrospective studies, the overall incidence of brain metastases (BM) in patients with NSCLC was 10–40% [2,3,4,5,6]. The prevalence of BM in patients with stage IV disease at presentation is approximately 26% [7]. The prognosis with BM is poor, and the median overall survival (OS) is generally 12 months or less [8]. To date, according to our knowledge, no study has looked at the survival benefit from detecting asymptomatic brain metastases in patients with NSCLC stage IV at presentation.

There are multiple considerations for the treatment of patients with BM. Chemotherapy has a limited role because of a presumed lack of effectiveness due to its poor penetration of the blood–brain barrier [9]. For patients presenting with epidermal growth factor receptor (*EGFR*)-mutated or anaplastic lymphoma kinase (*ALK*)-rearranged NSCLC, targeted therapies have shown efficacy and antitumor activity in the central nervous system (CNS) [10,11]. Additionally, emerging clinical data suggest that systemic immunotherapy monotherapy has activity in untreated BM from NSCLC with response rates around 30% [12,13,14,15]. Neurosurgery involves significant perioperative risks, and cranial irradiation is associated with toxicities, such as cognitive decline, radiation necrosis and cerebrovascular complications [16,17,18,19,20,21]. In the context of advanced disease, the objective of these treatments is palliation of symptoms.

In NSCLC, there are some discrepancies in practice guidelines over routine brain imaging for patients with advanced disease. The rationale behind routine neuroimaging in patients with stage IV disease would be early detection of brain metastases so that early treatment can be administered before development of neurologic deficits or seizures. Another possible benefit would be to treat less numerous and smaller lesions with stereotactic radiosurgery (SRS) and to avoid whole brain radiation therapy (WBRT). According to the American College of Chest Physicians (CHEST), brain imaging should be performed routinely on patients with stage III or IV disease, even if they do not have neurological symptoms [22]. The National Comprehensive Cancer Network (NCCN) also recommends that asymptomatic patients with stage IB or higher should undergo brain imaging [23]. The European Society of Medical Oncology (ESMO) claims that brain imaging should be reserved for patients with neurological symptoms [24]. In 2014, the *Institut national d’excellence en santé et en services sociaux* (INESSS) and the *Groupe d’étude en oncologie du Québec* (GEOQ) published algorithms for the investigation, treatment and monitoring of lung cancer. According to this document based on the opinion of fifty expert oncologists, a systematic investigation of asymptomatic brain metastases is recommended for stage III or higher [25]. The majority of practice guidelines advocate the use of magnetic resonance imaging (MRI) over computed tomography (CT) [22,23,24,25].

Given the lack of consensus in current practice guidelines, this study reports the proportion of patients with stage IV NSCLC without neurological symptoms undergoing brain imaging in their initial investigation, and survival outcomes according to initial cerebral imaging being performed or not.

## 2. Materials and Methods

### 2.1. Study Design

This retrospective study included all patients with stage IV NSCLC diagnosed between 1 January 2013 and 31 December 2016 at *Institut universitaire de cardiologie et de pneumologie de Québec* (IUCPQ). Patients were identified from the Oncology Database (SICTO). Patients with neurological symptoms at initial presentation were excluded.

### 2.2. Data Collection

Demographic data collected for this study included age, gender, smoking status and Eastern Cooperative Oncology Group performance status (ECOG PS). The medical charts were also reviewed for histopathological diagnosis, date of diagnosis, sites of metastases, use of a systemic treatment, *EGFR*, *ALK* and PD-L1 status. If brain imaging was performed initially at diagnosis or later in the course of the disease, the modality of imaging and the description of brain metastases were noted. Outcomes of patients were noted, mainly regarding the treatment modality offered for brain metastases, the onset of neurological symptoms or new/progressive brain metastases, and the date of death or last follow-up.

The primary outcome of this study was to compare the evolution of patients with stage IV NSCLC without neurological symptoms at diagnosis who had initial brain imaging with those who did not. Secondary outcomes were to determine the proportion of patients who underwent brain imaging in their initial investigation and the proportion of patients who developed metachronous CNS metastasis. The impact of mutational status on the presence of cerebral metastases and the course of the disease was also analyzed as a secondary objective.

### 2.3. Statistical Analysis

Patient demographics and clinical characteristics were summarized using descriptive methods. Continuous variables were reported as mean ± standard deviation (SD) and analyzed using Student’s *t*-test. Nominal variables were reported as frequencies and analyzed using the Fisher’s exact tests for comparisons between groups. For the OS of patients with and without imaging, the Kaplan–Meier estimates and the log-rank test for between-group comparisons were performed. A Gray’s test was used to account for death as a competing risk event for patients having metachronous CNS metastasis. For all statistical analyses, the results were considered significant with *p*-values < 0.05. Analyses were performed using SAS version 9.4 (SAS Institute Inc., Cary, North Carolina) and R package.

## 3. Results

### 3.1. Patients

**Figure 1:**
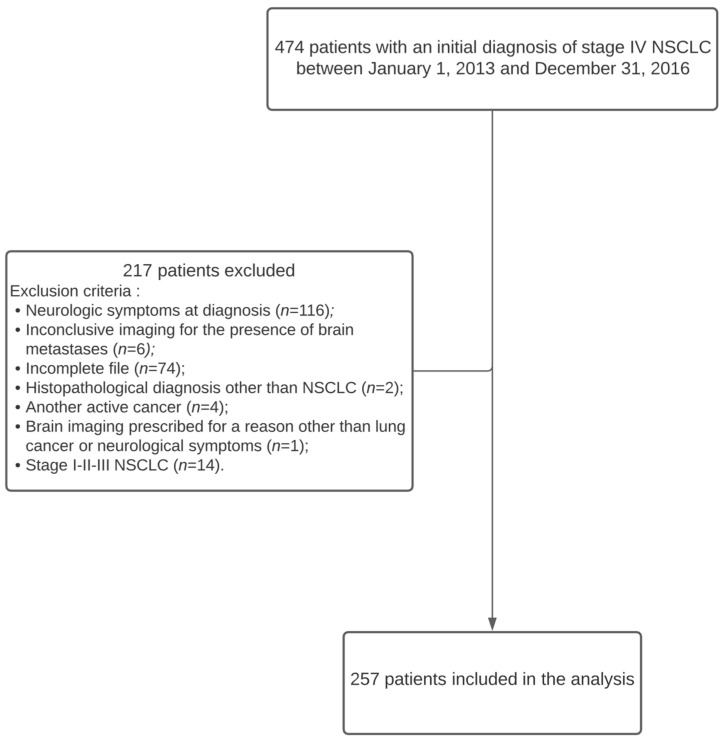
Consort diagram. NSCLC = non-small cell lung cancer.

Four hundred and seventy-four patients diagnosed with stage IV non-small cell lung cancer were identified from the Oncology Database (SICTO) between 1 January 2013 and 31 December 2016. Patients with neurologic symptoms at diagnosis (*n* = 116) were excluded. Furthermore, 74 patients were diagnosed at our center but were transferred back to their referring institution for treatment and lost to follow-up. Hence, these patients were excluded due to missing data. Twenty-seven patients were excluded for other various reasons, and, finally, 257 patients were included in the analysis (Figure 1).

### 3.2. Brain Imaging

Baseline characteristics of all patients are summarized in Table 1. The population was divided in two groups, with 145 patients who underwent initial CNS imaging and 112 patients who did not. Patients with initial imaging were younger than those without (median age 65.1 vs. 68.5, *p* = 0.0025). Most patients in both groups were male (56%), former or current smokers (95%) and had adenocarcinoma (73%). ECOG PS was often missing from the charts. *EGFR* and *ALK* alterations were uncommon (7% and 1%, respectively). Bones were the most common site of metastases, involved in 54% of subjects with CNS imaging versus 67% of those without (*p*= 0.04).

Among patients undergoing brain imaging, 112/145 (77%) patients had a CT scan, while MRI alone was chosen only in 18/145 (12%), and both imaging techniques were used in 15/145 (10%). CNS metastases were diagnosed in 47/145 patients (32%) with initial imaging (synchronous metastasis). Of these, 26/47 (55%) received systemic treatment. There was no difference in the proportion of patients receiving systemic therapy in the group with negative imaging (55/98, 56%) versus in the group without imaging (57/112, 51%).

### 3.3. Brain Metastasis

**Figure 2:**
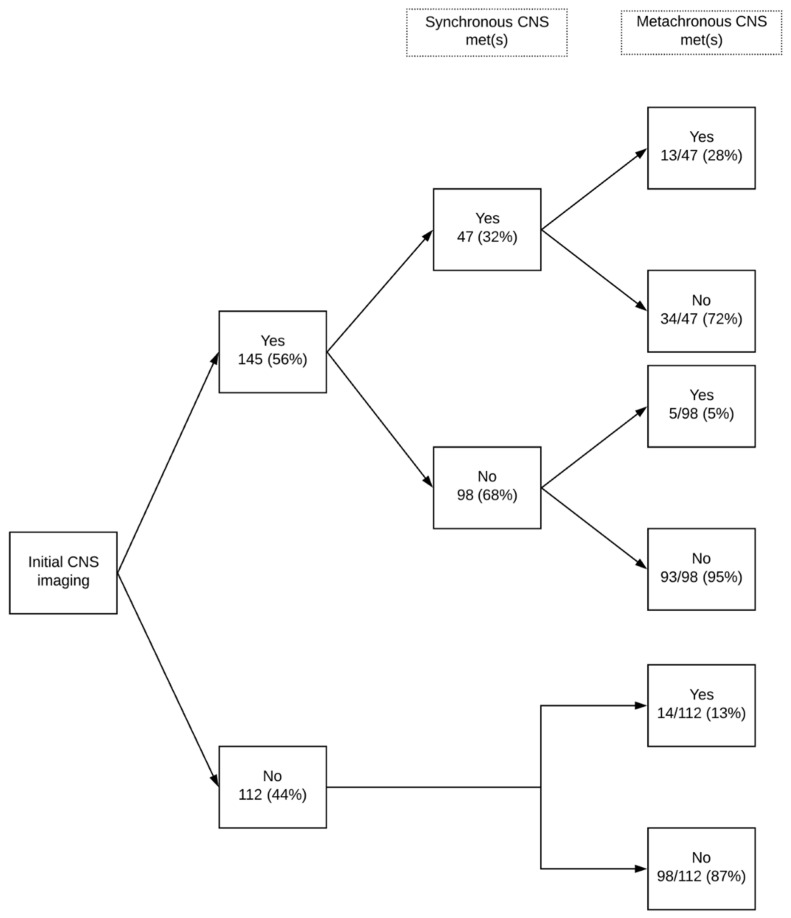
Synchronous and metachronous CNS metastases. CNS = central nervous system; met = metastasis.

Among patients with synchronous CNS metastasis, 18/47 (38%) had a single brain lesion and 11/47 (24%) had ≥5 (Table 2). Synchronous metastases were treated by WBRT in 25/47 (53%) patients, by SRS in 9/47 (19%) and by SRS and WBRT in 1/47 (2%), while 12/47 (26%) were not treated. Metachronous metastasis was diagnosed in 32 patients, including 13 patients with synchronous metastasis, 5 patients with initial negative imaging, and 14 patients without initial imaging (Figure 2). Only 5/32 were a single metachronous CNS metastasis. The majority of patients (28/32, 88%) were symptomatic. Metachronous metastases were treated by WBRT in 12/32 (38%) patients, by SRS in 5/32 (16%) and by surgery in 2/32 (6%), while 13/32 (41%) were not treated.

CNS imaging was performed at diagnosis in 11/17 (65%) patients with *EGFR*-mutated and in 3/3 (100%) with *ALK*-rearranged NSCLC. Synchronous CNS metastases were diagnosed in 1/17 (6%) and 2/3 (67%), respectively. These patients all received systemic therapy and underwent WBRT. *EGFR*-mutated and *ALK*-rearranged NSCLC were found to have metachronous CNS metastases in 4/17 (24%) and 1/3 (33%) of cases, and they were all symptomatic (Figures S1 and S2).

**Figure 3:**
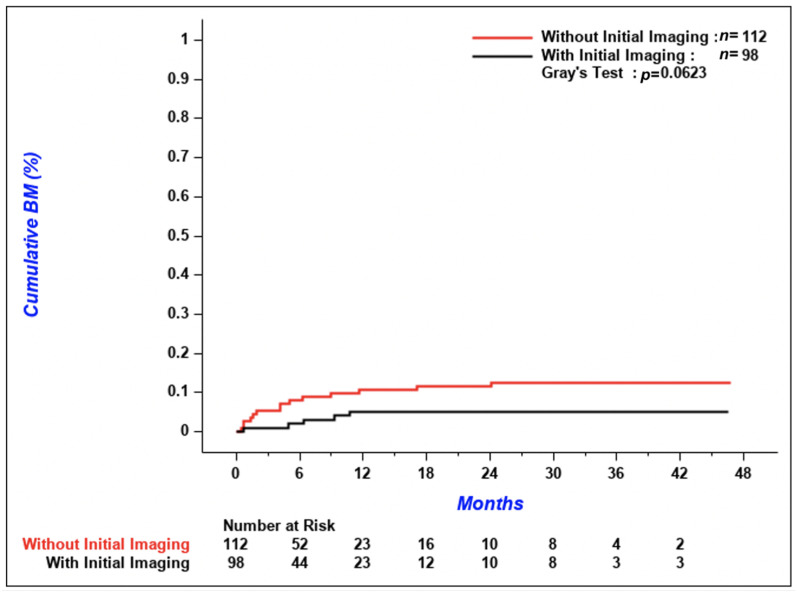
Cumulative incidence of metachronous brain metastases. BM = brain metastases.

**Figure 4:**
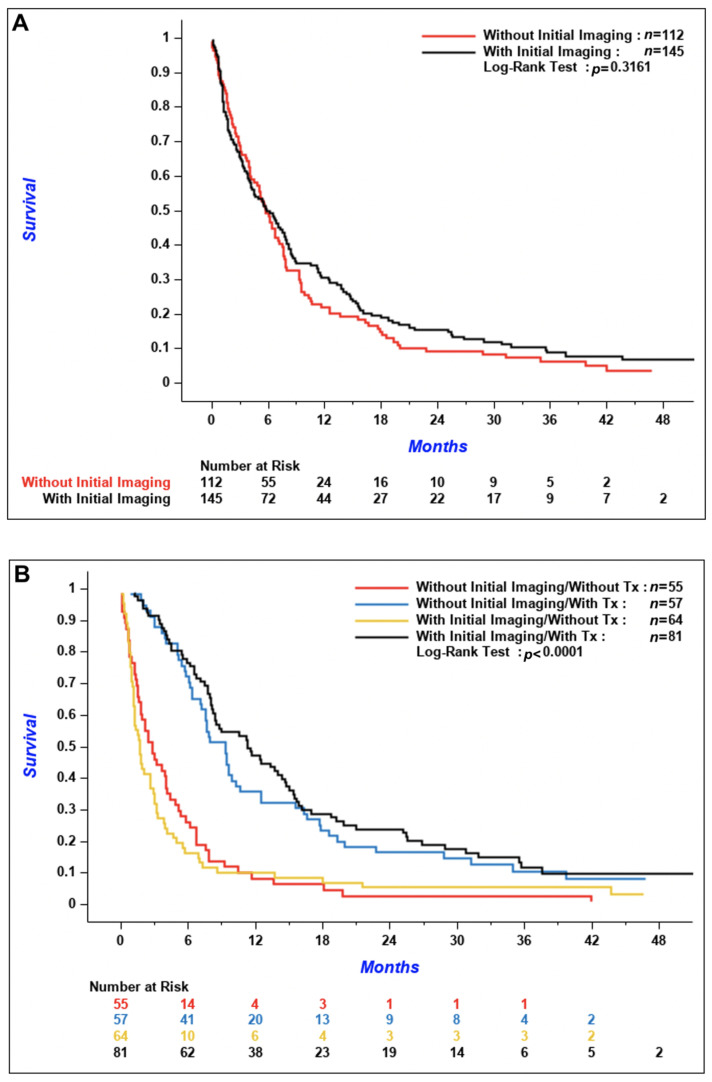
(**A**) Overall survival of patients with initial brain imaging versus patients without initial imaging. (**B**) Overall survival of patients with initial brain imaging versus patients without initial imaging, stratified by systemic treatment.

**Figure 5:**
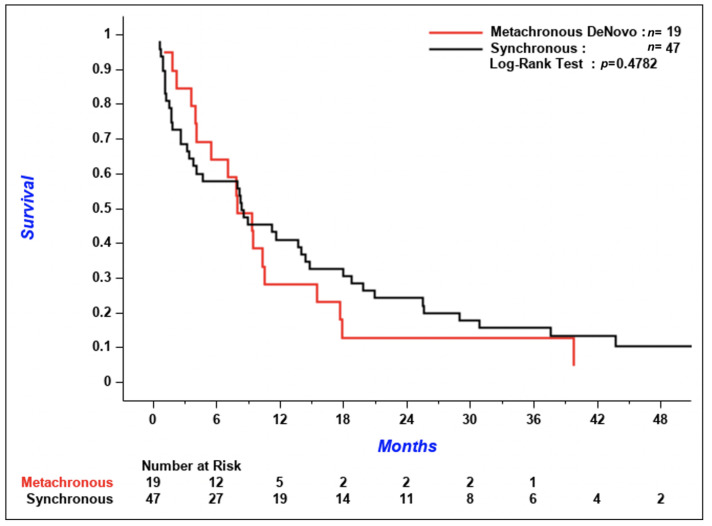
Time from diagnosis of CNS metastases to death in patients with synchronous CNS metastases versus de novo metachronous CNS metastases. CNS = central nervous system.

According to Figure 3, the cumulative incidence of metachronous CNS metastases tended to be lower in patients with initial imaging compared to patients without, but the difference between the two groups was not significant (*p* = 0.06). The 3-year cumulative incidence of brain metastases was 5.1% (95% confidence interval [CI] 1.9–10.8%) in patients with initial CNS imaging versus 12.5% (95% CI 7.1–19.4%) in patients without. Median OS was similar between the two groups; 5.9 months (95% CI 4.0–7.8) in patients with initial brain imaging and 5.8 months (95% CI 4.1–7.1) in patients without (Figure 4A, *p* = 0.32). According to Figure 4B, OS of patients who received systemic treatment was improved regardless of if initial brain imaging was performed or not (*p* < 0.001 for the comparison of patients who received systemic treatment versus patients who did not). There was no difference in the time from diagnosis of CNS metastases to death in patients with synchronous versus de novo metachronous metastases (Figure 5, *p* = 0.48).

## 4. Discussion

In this monocentric cohort of stage IV NSCLC patients without neurologic symptoms, CNS imaging, mainly with CT alone (77%), was performed at diagnosis in 56% of patients, and the prevalence of synchronous CNS metastasis among these patients was 32%. During the follow-up of the 257 patients in the entire cohort, 32 developed BM (metachronous metastasis). Among these 32 patients, 13 of them already had brain metastases initially. The prevalence of CNS metastases at any time during disease course in the whole cohort was 26%. We did not demonstrate a difference in median OS between patients who underwent initial CNS imaging and those who did not. OS of patients who received systemic treatment was improved regardless of if initial brain imaging was performed or not. However, we did show a tendency for a higher cumulative incidence of metachronous CNS metastasis in patients without baseline imaging, but the *p*-value was not statistically significant (*p* = 0.06). These metachronous metastases were symptomatic and were more often not treated when compared to synchronous metastases. However, there was no difference in the time from diagnosis of CNS metastases to death in patients with synchronous versus de novo metachronous metastases. The prevalence of patients with *EGFR*-mutated and *ALK*-rearranged NSCLC in our cohort was low, so we were not able to draw any conclusions on the impact of these biomarkers on the prevalence and incidence of brain metastases and on the course of the disease.

The question of our study is relevant. For patients without neurological symptoms, it is interesting to wonder if initial brain imaging changes the course of a disease already advanced at diagnosis. It must be established whether there is a survival benefit in diagnosing asymptomatic cerebral metastases in patients who already have a poor prognosis. The study design seems adequate to answer this question.

Regarding patient characteristics, the study sample still represents the usual population of patients with stage IV NSCLC. Median age of the patients was 67 years; they were predominantly male and former or current smokers. The majority had a histopathological diagnosis of adenocarcinoma, with a large tumor, multiple lymphadenopathies and extrathoracic metastases. The proportion of patients who have received systemic treatment and local therapies for brain metastases roughly reflects what is usually seen in practice.

### Limitations

Our results are limited by the retrospective nature of the study, by a small sample and by the fact that the study is unicentric. Several patients were excluded from the study because they had neurologic symptoms or were lost to follow-up. In addition, we clearly did not include enough patients with *ALK*-rearranged and *EGFR*-mutated NSCLC (*n* = 3 and 17 respectively) to assess the impact of these mutations on the presence of brain metastases and the course of their disease. This is partially explained by the fact that these mutations were not always routinely tested between 2013 and 2016, but also because our population in Quebec City is mainly composed of Caucasians and heavy smokers. Additionally, newer generation tyrosine kinase inhibitors (TKIs) were not available at that time, which might explain why baseline neuroimaging was not always performed in this population, and the high proportion of these patients undergoing WBRT, which is clearly not the treatment of choice in 2020. Given the retrospective nature of the study, some information were missing or poorly described, such as ECOG PS and symptoms. Furthermore, since the field of pulmonary oncology has evolved very quickly, it is unlikely that the trends described in this study can be transposed to the past few years.

## 5. Conclusions

In this retrospective cohort of patients with stage IV NSCLC without neurologic symptoms, there was no significant difference in terms of survival in the group who underwent initial brain imaging compared to the group who did not. OS of patients seemed to be influenced more by systemic treatment rather than initial brain imaging. However, the cumulative incidence of metachronous brain metastasis tended to be lower in patients with initial brain imaging.

More studies are needed to assess the effects on various outcomes (incidence of metachronous CNS metastases, development of symptoms, choice of local or systemic treatments, median OS) of routine neuroimaging in asymptomatic patients with stage IV NSCLC, with ideally prospective cohorts who have access to contemporary treatments, including immunotherapy, newer generation TKIs and SRS. We still believe there is a rationale for early detection of brain metastases so that early treatment can be administered before the development of neurologic deficits or seizures.

## Figures and Tables

**Table 1:** Patient characteristics.

	All Patients	Patients Who Underwent Initial CNS Imaging	Patients Who Did Not Underwent Initial CNS Imaging	*p*-Value
	*n* = 257	*n* = 145	*n* = 112	
Median age, years ± SD	66.6 ± 9.2	65.1 ± 9.5	68.5 ± 8.4	0.0025
Male sex	144 (56%)	76 (52%)	68 (61%)	0.2059
Former or current smoker	244 (95%)	140 (97%)	104 (93%)	0.2514
ECOG PS				
0–1	70 (27%)	41 (28%)	29 (26%)	0.5612
≥2	40 (16%)	25 (17%)	15 (13%)	
Not reported	147 (57%)	79 (55%)	68 (61%)	
Histology				
Adenocarcinoma	187 (73%)	106 (73%)	81 (72%)	0.6169
Squamous carcinoma	44 (17%)	25 (17%)	19 (17%)	
Adenosquamous carcinoma	5 (2%)	4 (3%)	1 (1%)	
Large cell carcinoma	1 (0%)	1 (1%)	0	
NSCLC NOS	20 (8%)	9 (6%)	11 (10%)	
*EGFR* mutation				
Positive	17 (7%)	11 (8%)	6 (5%)	0.8239
Negative	153 (59%)	85 (59%)	68 (61%)	
Not tested (squamous carcinoma)	43 (17%	25 (17%)	18 (16%)	
Not tested (*ALK-*positive)	3 (1%)	3 (2%)	0	
Unknown*	41 (16%)	21 (14%)	20 (18%)	
*ALK* rearrangement				
Positive	3 (1%)	3 (2%)	0	0.3685
Negative	171 (66%)	97 (67%)	74 (66%)	
Not tested (squamous carcinoma)	43 (17%)	25 (17%)	18 (16%)	
Unknown *	40 (16%)	20 (14%)	20 (18%)	
PD-L1 status				
<1%	6 (2%)	5 (4%)	1 (1%)	0.5636
1–49%	7 (3%)	3 (2%)	4 (4%)	
≥50%	12 (5%)	7 (5%)	5 (5%)	
Unknown **	232 (90%)	130 (90%)	102 (91%)	
T stage				
1	28 (11%)	14 (10%)	14 (13%)	0.2783
2	53 (21%)	31 (21%)	22 (20%)	
3	61 (24%)	33 (23%)	28 (25%)	
4	94 (37%)	59 (41%)	35 (31%)	
x	21 (8%)	8 (6%)	13 (12%)	
N stage				
0	28 (11%)	15 (10%)	13 (12%)	0.1104
1	20 (8%)	13 (9%)	7 (6%)	
2	97 (38%)	46 (32%)	51 (46%)	
3	105 (41%)	68 (47%)	37 (33%)	
x	7 (3%)	3 (2%)	4 (4%)	
Sites of metastases at diagnosis				
CNS	47 (18%)	47 (32%)		
Lung	54 (21%)	30 (21%)	24 (21%)	0.8788
Pleura	37 (14%)	18 (12%)	19 (17%)	0.3707
Liver	69 (27%)	34 (24%)	35 (31%)	0.2013
Bone	153 (59%)	78 (54%)	75 (67%)	0.0403
Adrenal	61 (24%)	30 (21%)	31 (38%)	0.2369
CNS imaging modality				
CT		112 (77%)		
MRI		18 (12%)		
Both		15 (10%)		

ALK = Anaplastic lymphoma kinase; CNS = central nervous system; CT = computed tomography; ECOG PS = Eastern Cooperative Oncology Group performance status; EGFR = epidermal growth factor receptor; MRI = magnetic resonance imaging; NOS = not otherwise specified; NSCLC = non-small cell lung cancer; SD = standard deviation. * Testing was not performed for 40 patients for various reasons: poor performance status precluding systemic treatment (*n* = 27), treatment refusal by patient (*n* = 5), lack of tissue (*n* = 4) and no reason documented in the medical chart (*n* = 4). Additionally, one patient had an *ALK*-negative tumor but insufficient tissue for *EGFR* testing. ** PD-L1 status is unknown in the majority of cases since immune checkpoint inhibitors were not readily available at this time.

**Table 2:** CNS metastasis.

	Synchronous CNS Metastasis	Metachronous CNS Metastasis			*p*-Value *
		With CNS Metastasis at Initial Imaging *	Without CNS Metastasis at Initial Imaging	Without Initial Imaging	
	*n* = 47	*n* = 13	*n* = 5	*n* = 14	
Number of metastases					
1	18 (38%)	2 (15%)	2 (40%)	1 (7%)	0.1024
2–4	18 (38%)	5 (39%)	0	8 (57%)	
≥5	11 (23%)	6 (46%)	3 (60%)	5 (36%)	
Symptoms					
None	47 (100%)	4 (31%)	0	0	<0.0001
Headache		3 (23%)	0	3 (21%)	
Neurological impairement		4 (31%)	5 (100%)	11 (79%)	
Seizure		2 (15%)	0	0	
Treatment					
None	12 (26%)	4 (31%)	2 (40%)	7 (50%)	0.5704
WBRT	25 (53%)	4 (31%)	2 (40%)	6 (43%)	
SRS	9 (19%)	3 (23%)	1 (20%)	1 (7%)	
SRS + WBRT	1 (2%)	0	0	0	
Surgery	0	2 (15%)	0	0	

CNS = central nervous system; SRS = stereotactic radiosurgery; WBRT = whole brain radiotherapy. * The *p*-values do not apply for patients with metachronous CNS metastasis who already had CNS metastasis at initial imaging (*n* = 13).

## Data Availability

The data presented in this study are available on request from the corresponding author.

## Supplementary Materials

The following are available online at https://www.mdpi.com/1718-7729/28/2/108/s1, Figure S1: Synchronous and metachronous CNS metastases (*EGFR*-mutated NSCLC), Figure S2: Synchronous and metachronous CNS metastases (*ALK*-rearranged NSCLC).
