# Supplementary material for: Routine Neuroimaging in Patients with Stage IV Non-Small Cell Lung Cancer: A Single Center Experience
Source: Curr Oncol. 2021 Mar 2;28(2):1125–36. doi: 10.3390/curroncol28020108 (PMC8025755; doi:10.3390/curroncol28020108)
Supplement: Supplementary file 1 [file curroncol-28-00108-s001.pdf]

Supplementary Materials

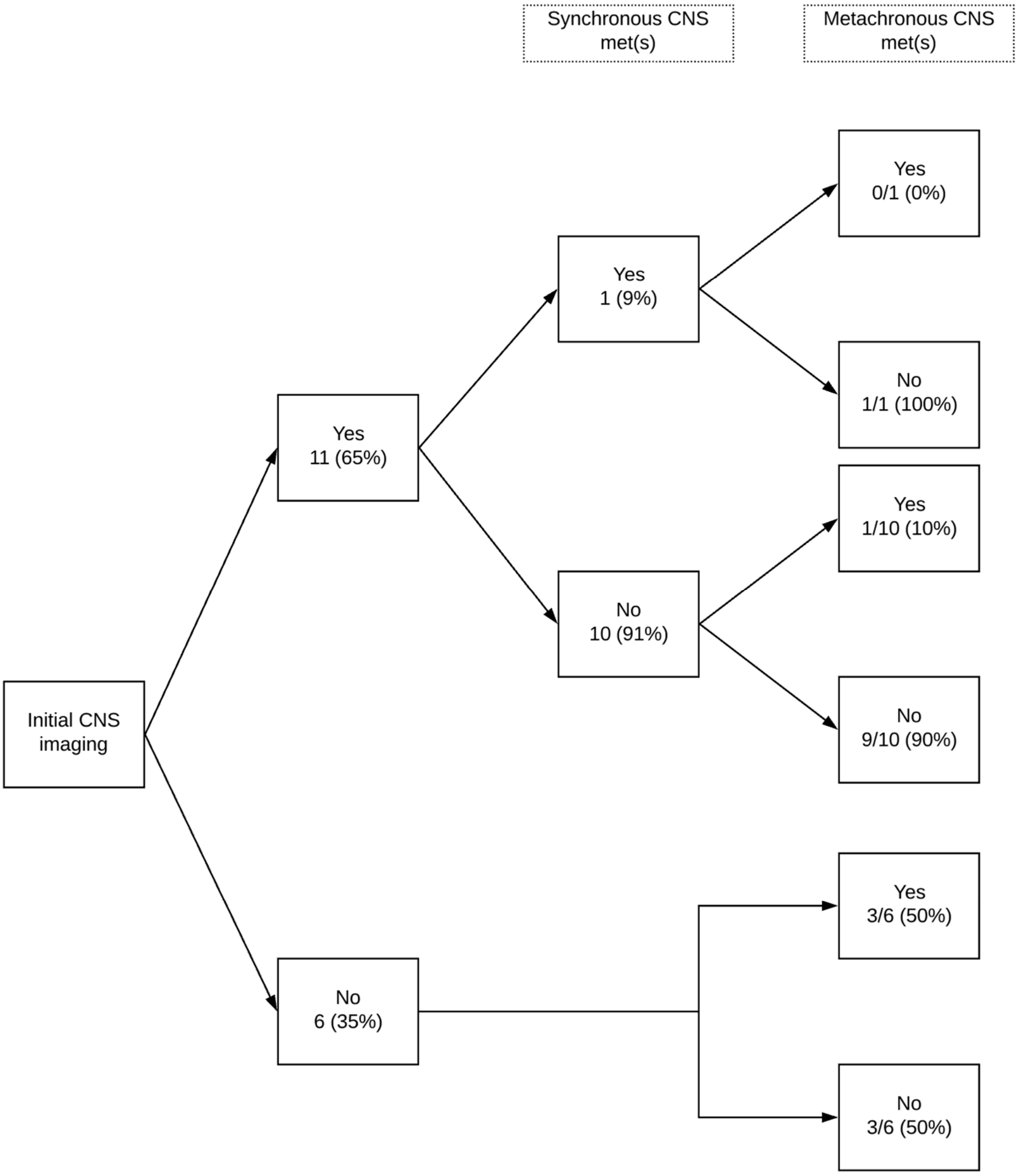

Figure S1. Synchronous and metachronous CNS metastases (*EGFR*-mutated NSCLC).

CNS = central nervous system; EGFR = epidermal growth factor receptor; met = metastasis; NSCLC = non-small cell lung cancer.

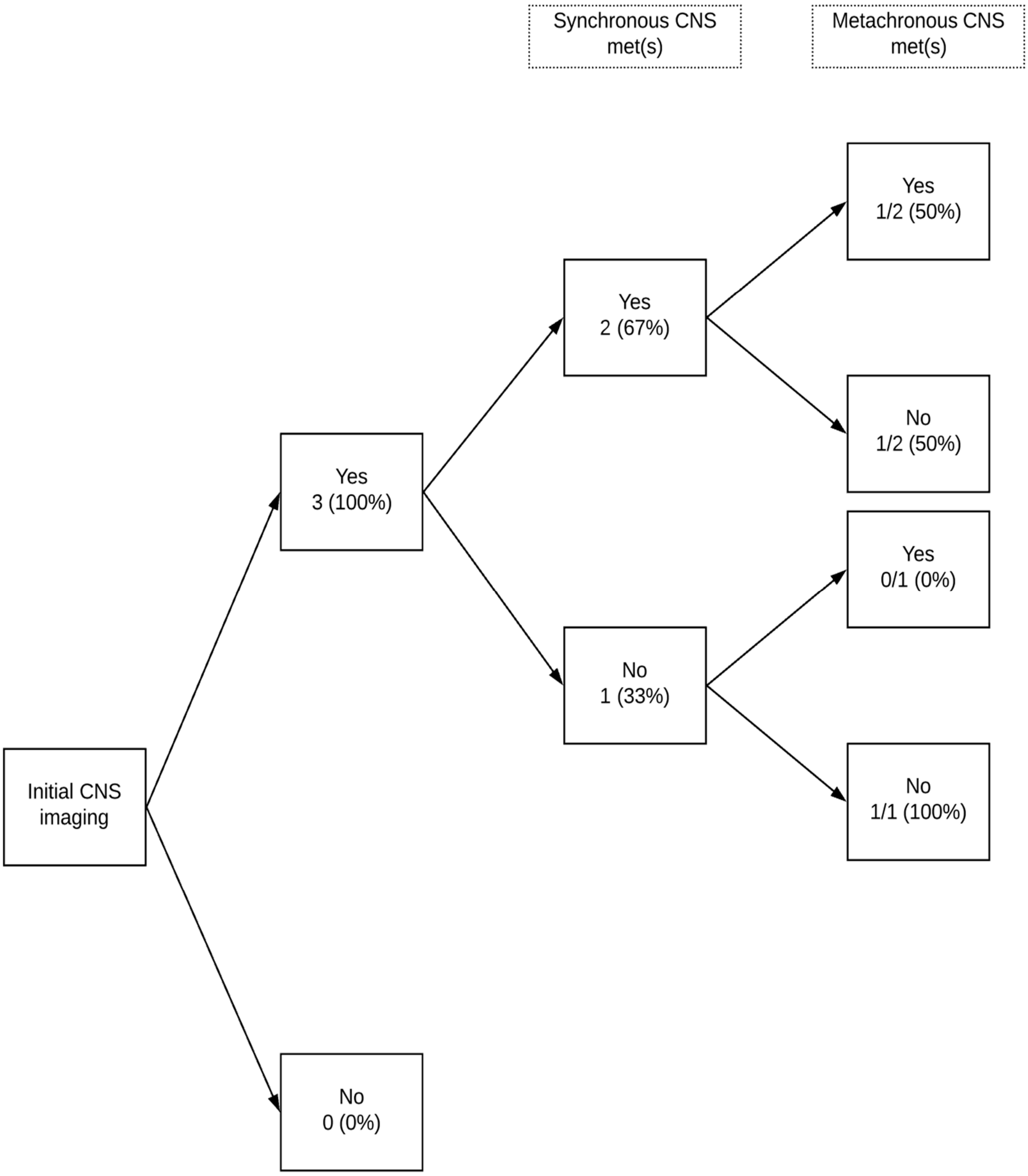

Figure S2. Synchronous and metachronous CNS metastases (*ALK*-rearranged NSCLC).

ALK = anaplastic lymphoma kinase; CNS = central nervous system; met = metastasis; NSCLC = non-small cell lung cancer.
